# Porcine Decellularized Diaphragm Hydrogel: A New Option for Skeletal Muscle Malformations

**DOI:** 10.3390/biomedicines9070709

**Published:** 2021-06-22

**Authors:** Daniele Boso, Eugenia Carraro, Edoardo Maghin, Silvia Todros, Arben Dedja, Monica Giomo, Nicola Elvassore, Paolo De Coppi, Piero Giovanni Pavan, Martina Piccoli

**Affiliations:** 1 Fondazione Istituto di Ricerca Pediatrica Città della Speranza, Corso Stati Uniti 4, 35127 Padova, Italy; d.boso@irpcds.org (D.B.); eugenia.carraro@studenti.unipd.it (E.C.); edoardomaghin@gmail.com (E.M.); piero.pavan@unipd.it (P.G.P.); 2 Department of Industrial Engineering, University of Padova, Via Venezia 1, 35131 Padova, Italy; silvia.todros@unipd.it; 3 Department of Biomedical Sciences, University of Padova, Via Ugo Bassi 58/B, 35131 Padova, Italy; 4 Department of Cardiac, Thoracic, Vascular Sciences and Public Health, University of Padova, Via Giustiniani 2, 35128 Padova, Italy; arben.dedja@unipd.it; 5 Department of Industrial Engineering, University of Padova, Via Marzolo 9, 35131 Padova, Italy; monica.giomo@unipd.it (M.G.); nicola.elvassore@unipd.it (N.E.); 6 Veneto Institute of Molecular Medicine, Via G. Orus 2, 35127 Padova, Italy; 7 Shanghai Institute for Advanced Immunochemical Studies (SIAIS), ShanghaiTech University, Y Building, No. 393 Middle Huaxia Road, Pudong, Shanghai 201210, China; 8 NIHR Biomedical Research Center, Great Ormond Street Institute of Child Health, University College London, London WC1N 1EH, UK; p.decoppi@ucl.ac.uk; 9 Specialist Neonatal and Pediatric Surgery, Great Ormond Street Hospital, London WC1N 3JH, UK

**Keywords:** tissue engineering, skeletal muscle, extracellular matrix, hydrogel, diaphragmatic hernia

## Abstract

Hydrogels are biomaterials that, thanks to their unique hydrophilic and biomimetic characteristics, are used to support cell growth and attachment and promote tissue regeneration. The use of decellularized extracellular matrix (dECM) from different tissues or organs significantly demonstrated to be far superior to other types of hydrogel since it recapitulates the native tissue’s ECM composition and bioactivity. Different muscle injuries and malformations require the application of patches or fillers to replenish the defect and boost tissue regeneration. Herein, we develop, produce, and characterize a porcine diaphragmatic dECM-derived hydrogel for diaphragmatic applications. We obtain a tissue-specific biomaterial able to mimic the complex structure of skeletal muscle ECM; we characterize hydrogel properties in terms of biomechanical properties, biocompatibility, and adaptability for in vivo applications. Lastly, we demonstrate that dECM-derived hydrogel obtained from porcine diaphragms can represent a useful biological product for diaphragmatic muscle defect repair when used as relevant acellular stand-alone patch.

## 1. Introduction

Tissue engineering approaches have been further developed and refined during the last years in order to produce organ and tissue substitutes for the treatment of several diseases [1,2,3,4]. An abundance of studies highlighted the opportunity that hydrogels offer as 3D scaffolds for advanced in vitro cell cultures and in vivo tissue replacement [5,6,7,8]. Among the different types of hydrogel that have been developed (synthetic, natural, or mixed hydrogels), decellularized extracellular matrix (ECM)-derived formulations are considered nowadays the best choice to mimic the tissue structure and composition because they consist of a natural microenvironment that is devoid of cells and that facilitates reseeded cell–ECM connections and 3D cellular organization in a similar manner to those of living tissues [9]. As for other organs, decellularized ECM (dECM) from skeletal muscle (SKM) can be transformed into hydrogel, exploiting a collagen-based self-assembly process that is regulated in part by the presence of glycosaminoglycans, proteoglycans, and other ECM proteins [10]. Several authors employed this technology for producing SKM dECM-derived hydrogels, which can be used in different ways such as cell-laden constructs with 3D-bioprintable characteristics or for improved in vivo cell delivery in volume muscle loss model [11,12,13]. Each specific application requires different parameters to be taken into account. Biomaterial porosity and its ability to be remodeled by loaded cells are pivotal for cellular encapsulation, since these characteristics favor cell survival, proliferation, and differentiation [14]. On the other hand, when hydrogels are used as filling material, especially in organs in which mechanical stress is significant, as in the SKM, control of the degradation rate and biomaterial mechanical properties are determining factors. In this latter situation, a variety of crosslinking strategies (chemical, physical, or with laser light) have been developed to increase hydrogel stiffness and strength with the aim of generating a substitute with the desired biomechanical characteristics [15,16]. 

Among all SKM defects and malformations, congenital diaphragmatic hernia (CDH) still represents a challenge for clinicians due to the high mortality rate and the important side effects that come as a consequence of using synthetic patches to repair the defect, which are currently the gold standard for the treatment of CDH [17,18,19]. In the last decades, biological substitutes have been engineered to provide a response to these issues. However, the clinical use of these products, which are obtained from tissues different from SKM, did not demonstrate a clear improvement compared to synthetic materials [20,21]. It is important to note that the diaphragm is a complex and unique tissue with specific physiological characteristics [22,23]; our group demonstrated that the use of a biologic patch obtained directly from the decellularization of diaphragmatic muscle considerably improved the treatment of CDH in an animal model [24], thus confirming the necessity of using a tissue-specific biomaterial to obtain a superior treatment for CDH. Unfortunately, donor availability and large-scale production are limiting factors to the implementation of this approach in clinical practice. Therefore, the manufacturing of a standardized and ready-to-use biomaterial specific for diaphragmatic muscle repair could be a winning strategy to address the difficulties of biologic material tracing and clinical management of this disease.

Herein, to the best of our knowledge, we developed and characterized the first porcine diaphragmatic dECM-derived hydrogel for diaphragm applications. We obtained a tissue-specific biomaterial able to mimic the complex structure of SKM ECM, we determined hydrogel biocompatibility and biomechanical aptitude for in vivo application, and we demonstrated that dECM-derived hydrogels obtained from porcine diaphragm represent a useful biological product to use as tissue patches for the treatment of diaphragmatic malformations, such as CDH.

## 2. Materials and Methods

### 2.1. Decellularization, Lyophilization, and Pulverization of Diaphragmatic Extracellular Matrix-Derived Muscle

Piglet (*Sus scrofa domesticus*) muscles (*n* = 5) purchased from the food market were either processed immediately and decellularized or stored at −80 °C until ready to use. If frozen, samples were first thawed at +4 °C for at least 6 h before decellularization. Once defrosted and before decellularization, native diaphragms were sectioned in small pieces (0.7 g), cleaned of fat and connective tissue, disinfected with betadine 10% for 30 min, washed several times in 1× sterile phosphate-buffered saline (PBS, Thermo Fisher Scientific, MB, Milan, Italy) and then transferred to deionized water in order to start the decellularization process. Samples were processed with four detergent-enzymatic treatment (DET) cycles to obtain complete cell removal. Each DET cycle comprised an incubation in deionized water at 4 °C for 24 h, 4% sodium deoxycholate (Sigma, Merck KGaA, Darmstadt, Germany) at room temperature (RT) for 4 h with gentle agitation, and 2000 kU DNase-I (Sigma, Merck KGaA, Darmstadt, Germany) in 1 M NaCl (Sigma, Merck KGaA, Darmstadt, Germany) at RT for 3 h. After decellularization, matrices were washed for at least 3 days with deionized sterile water, lyophilized with a −50 °C vacuum freeze-drying instrument (FD-1 A-50, Beijing Boyikang Instrument Co., LTD, Shanghai, China), and finally grinded to fine powder and stored at RT or −20 °C.

### 2.2. DNA Amount Quantification

To assess total DNA content within the native and decellularized diaphragms, specimens were extracted using DNeasy Blood & Tissue kits (Qiagen, Hiden, Germany) according to manufacturer’s instruction. DNA sample contents were then quantified using a Nanodrop 2000 spectrophotometer (Thermo Scientific, Waltham, MA, USA).

### 2.3. Mass Spectrometry

About 5 mg of 3 lyophilized samples were analyzed in triplicate (9 total specimens), resuspended in 0.2 mL 50 mM ammonium bicarbonate buffer and digested according to the protocol published by Naba et al. [25]. In brief, samples were reduced, alkylated, deglycosylated (PNGaseF) and digested twice with Lys-C and Trypsin enzymes. Digested peptides were dried under vacuum and stored at −20 °C until analysis. Digested samples were analyzed with a UHPLC-XEVO-G2-XS (Waters, Etten-Leur, The Nederland) mass spectrometer. The peptide mixtures were separated with a Biobasic C18 column, 5 μm, using a 3–45% linear gradient of CH_3_CN + 0.1% trifluoroacetic acid (TFA) (mobile phase B) in H_2_O + 0.1% TFA (mobile phase A) over a 110 min long analysis. Mass spec data were acquired in data-dependent mode in the 350–2000 m/z mass range. Instrumental parameters were set as follows: source: ESI (+); precursor charge selection: from 2 to 4; resolution: 22,000. Mass spec data were lock-mass corrected, peak picked, converted into mzML format and processed by Proteome Discoverer 2.2 (ThermoFisher Scientific, Monza, Italy) using the Sequest HT algorithm for protein identification. Search parameters were set as follows: database, UniprotKB reference proteome AUP000008227; enzyme, Trypsin (max 2 missed cleavages); taxonomy, *Sus scrofa*; precursor mass tolerance, 25 ppm, fragment mass tolerance, 0.08 Da. Fixed modifications: carbamidomethyl (C). Dynamic modifications: oxidation (M, P, K); deamidation (N, Q), and phosphorylation (S, T, Y). An acceptable protein false discovery rate (FDR) was set at <0.01 and a minimum of 2 non-redundant peptides were used for proteins identification.

### 2.4. Decellularized ECM-Derived Hydrogel Preparation

A three-step protocol described by the Choi group [11] was followed to prepare decellularized ECM-derived hydrogels. Lyophilized powder (1, 2, 3% w/v) was enzymatically digested in a solution containing pepsin 10% w/w (Sigma, Merck KGaA, Darmstadt, Germany) in 0.5 M acetic acid, for 48 h under agitation. To restore osmolarity and physiological conditions of the acidic solution, pH was adjusted to the physiological value of 7.4 and a further 10% v/v 1× PBS was added. Temperature-induced gelation of the neutralized solution was achieved by keeping the pre-gel solution at 37 °C degrees until spontaneous formation of collagen fibrils occurred and hydrogels were formed. The natural molecule genipin (Sigma, Merck KGaA, Darmstadt, Germany) was used to induce the crosslinking process in the hydrogels. Immediately after the formation of hydrogels, a solution containing 1 mM genipin in 1× PBS was added and kept for overnight incubation. The higher the crosslinking, the more intense was the blue coloring of the hydrogels; lastly, the hydrogels were washed three times in 1× PBS and subsequently maintained in 1× PBS until ready for analysis or experiments.

### 2.5. Scanning Electron Microscopy (SEM) and Mean Pore Size Measurement

Hydrogels were rinsed with warm 1× PBS overnight, sliced into segments of approximately 0.5 cm and fixed with 3% glutaraldehyde (Sigma, Merck KGaA, Darmstadt, Germany) in 0.1 M phosphate buffer at RT overnight. After fixation and PBS washes, the samples were dehydrated in a series of graded ethanol–water mixtures from 15 to 100% ethanol, critical point dried using CO_2_, and mounted on aluminum stubs using sticky carbon taps. Hydrogels were mounted and coated with a thin layer of Au/Pd (approximately 2 nm thick) using a Gatan ion beam coater. Images were recorded with a JEOL JSM 6490 scanning electron microscopy. Porosity and mean pore size were calculated using the ImageJ software plugin Diameter J.

### 2.6. Fluorescence Recovery after Photobleaching (FRAP)

Fluorescence recovery after photobleaching measurements were carried out on a Leica SP5 confocal microscope, equipped with an Argon laser 488 nm, 30 mW nominal power. A 10× AIR objective with a digital magnification of 4× was used for all experiments.

Hydrogels were first immersed in fluorescein isothiocyanate (FITC)–dextran average molecular weight 150,000 Da (Sigma, Merck KGaA, Darmstadt, Germany; data not shown) or 500,000 Da (Sigma, Merck KGaA, Darmstadt, Germany) solutions prepared in PBS, and incubated at RT for 20 min. Probe concentration was 2 mg/mL, a value that was chosen to give a linear response in fluorescence versus concentration. Experiments were performed at 90% of laser power, with a scanning frequency of 600 Hz. The AOTF (acousto-optic tunable filter) was set to 100% to obtain maximal bleaching. Recovery images were recorded with an AOTF setting of 13% before and after photobleaching. For each sample, at least three regions were investigated. Fluorescence recovery was measured at the center of the bleached area (round region of interest (ROI) 30 μm in diameter) and data were exported as Excel files. For each collected data set, the integrated fluorescence over the disk at any time t, *I(t)*, was normalized by its unbleached value, *I_pre_*, recorded prior the photobleaching of the disk. The resulting recovery curve, $f\left(t\right)=I\left(t\right)/I_{pre}$, was fitted using the Uniform Disk Model (UDM) derived by Braeckmans [26]

(1) $$\frac{F\left(t\right)}{F_{0}}=1-K_{0}\left[1-e^{-\varepsilon }\left(I_{0}\left(\varepsilon \right)+I_{1}\left(\varepsilon \right)\right)\right]$$

where *ε* is the grouped parameter:

(2) $$\varepsilon =\frac{w^{2}}{2\;Dt}$$

and *F* is the fluorescence in the disk at time *t*, *F*_0_ is the fluorescence in the disk before the photobleaching, *K*_0_ is a photobleaching parameter, *I*_0_ and *I*_1_ are the modified Bessel functions of zeroth and first order, respectively, *w* is the radius of the disk, *D* is the diffusion coefficient of the dextran. Moreover, to account the immobile fraction of molecules, the following relationship was used:

(3) $$F\left(t\right)=k\frac{F\left(t\right)}{F_{0}}-\left(1-k\right)\frac{F\left(0\right)}{F_{0}}$$

where *k* represents the mobile fraction and (1 − *k*) is the immobile fraction.

The diffusion coefficient, *D*, and the experimental parameters *k* and *K*_0_ were determined by a generalized reduced gradient (GRG) nonlinear least-square fit of Equations (1)–(3) to the experimental recovery curves.

### 2.7. Sample Preparation and Immunofluorescence Analysis

Hydrogels were washed three times with 1× PBS, fixed with paraformaldehyde (PFA) at RT, and embedded in optimal cutting temperature (OCT) compound. For immunofluorescence analysis, tissue slides were permeabilized with a solution containing 0.5% Triton X-100 (Fluxa, Milan, Italy), 1% bovine serum albumin (BSA, (Sigma, Merck KGaA, Darmstadt, Germany) in 1× PBS for 10 min at RT. Samples were then rinsed in 1× PBS for 10 min and blocked with 3% BSA, 0.1% Triton X-100 in 1× PBS overnight at 4 °C. After a washing step in 1× PBS for 10 min, samples were incubated with primary antibodies (listed in Table S2) diluted in 1% BSA, 0.1% Triton X-100 in 1× PBS for 1 h at 37 °C, and subsequently labeled with the appropriate secondary antibody (listed in Table S2) for 1 h at 37 °C. Nuclei were counterstained with fluorescent mounting medium plus (100 ng/mL) 4′,6-diamidino-2-phenylindole (DAPI; Sigma, Merck KGaA, Darmstadt, Germany).

### 2.8. Collagen and Hyaluronic Acid (HA) Quantification

For soluble and insoluble collagen quantifications, 15 mg of either wet native tissue or decellularized tissue, powder, and hydrogels was digested overnight with a solution containing 0.1 mg/mL pepsin in 0.5 M acetic acid. Soluble collagens in the supernatant were quantified through Sircol Soluble Collagen Assay (Biocolor, Carrickfergus, UK) following manufacturer instructions, whereas residual tissue from cold-acid extraction were fragmented and insoluble collagens were quantified with S2000 Sircol Insoluble collagen assay (Biocolor, Carrickfergus, UK). For hyaluronic acid (HA) quantification, 100 mg of tissue was digested overnight with Proteinase K (Sigma, Merck KGaA, Darmstadt, Germany) and HA quantified through Purple-Jelley Hyaluronan assay (Biocolor, Carrickfergus, UK).

### 2.9. Rheometric Analysis

Hydrogel rheological properties were measured at 37 °C by means of the rheometer Discovery HR 10 (TA instruments, New Castle, DE, USA), using 25 mm plate geometry. Hydrogels were placed on a plate at 37 °C for 30 min prior to measuring their mechanical properties. Hydrogel viscosity was evaluated by using the steady shear sweep mode with a shear rate ranging from 0.1 to 100 s^−1^. For rheological analysis in oscillatory temperature ramp, (1 mL) neutralized pre-gel solution was placed on the plate, a constant angular frequency of 6 rad/s was applied, and temperature was progressively increased with a ramp from 5–37 °C for 15 min followed by a constant temperature of 37 °C until 70 min. Storage (G’) and loss (G’’) moduli were measured for the entire period.

### 2.10. Turbidimetric Gelation Kinetics

ECM hydrogel gelation kinetics were evaluated by means of turbidimetric analysis, according to experimental methods already reported by several studies [27,28,29]. Optical density was measured at 405 nm from 100 μL samples of neutralized dECM of three different batches in triplicate (total amount of 9 samples) every 5 min for up to 2 h using a pre-heated to 37 °C UV-visible spectrophotometer (TECAN Spark Control, Mannedorf, Switzerland). Mean absorbance values and standard error of the mean (s.e.m.) were calculated from test repetitions at each time point. Normalized absorbance ($A_{n}$) was determined by the following equation:

(4) $$A_{n}\left(t\right)=\frac{A\left(t\right)-A_{0}}{A_{max}-A_{0}}$$

where $A\left(t\right)$ is the absorbance measured at a specific time point, $A_{0}$ is the initial absorbance, and $A_{max}$ is the maximum absorbance. Gelation kinetics parameters were calculated from experimental curves: the time to half gelation $\left(t_{1/2}\right)$ was defined as the time when 50% of the maximum absorbance value is measured, the gelation rate (*S*) was defined as the slope of the linear region of the gelation curve, and the lag time ($t_{lag}$) was defined as the intercept of the line with S slope with 0% absorbance.

### 2.11. Enzymatic Degradation Assay

Hydrogels were formed starting from 100 μL of pre-gel solution and rinsed in 1× PBS for several hours. PBS was removed and replaced with a solution containing 0.1% (*w/v*) Collagenase I from *Clostridium histolyticum* (Sigma, Merck KGaA, Darmstadt, Germany) in DMEM 1 g/L glucose (+) L-glutamine (+) pyruvate (Gibco-Fischer Scientific, Milan, Italy) and maintained at 37 °C for 120 min. To monitor the degradation rate over time, a picture of each hydrogel was acquired every 5 min, and hydrogel areas calculated with ImageJ at each time point. Remaining area (%) was calculated at each time point as a percentage of the initial area of the hydrogel. Controls were hydrogels kept in 1× PBS for the entire length of the experiment.

### 2.12. Cell Culture and Maintenance

Human skeletal muscle cells (hSKMCs, Gibco-Fischer Scientific, Milan, Italy) were grown in proliferative medium composed of 20% FBS (Gibco-Fischer Scientific, Milan, Italy), 10^−6^ M dexamethasone (Sigma, Merck KGaA, Darmstadt, Germany), 10 ng/mL basic fibroblast growth factor (bFGF) (R&D System, Minneapolis, Minnesota, USA), 10 μg/mL insulin (Gibco-Fischer Scientific, Milan, Italy) and 1% pen/strep (Gibco-Fischer Scientific, Milan, Italy) in DMEM low glucose (1 g/L D-glucose, Gibco-Fischer Scientific, Milan, Italy). Human dermal fibroblasts (hFbs, ATCC) were cultured in DMEM high glucose (4.5 g/L D-glucose, Gibco-Fischer Scientific, Milan, Italy) supplemented with 20% FBS, 1% L-glutamine (Gibco-Fischer Scientific, Milan, Italy) and 1% pen/strep. hSKMCs and hFbs were separately expanded until passage 9 at 37 °C, 5% CO_2_ with oxygen tension of 10% in humidified chamber.

### 2.13. Ex Vivo CDH

Mouse diaphragms (total *n* = 7) were harvested and collected in milli-Q H_2_O, according to protocol number 418/2020-PR. Each fresh sample was manually sutured to a home-made polydimethylsiloxane (PDMS) o-ring using a 6-0 silk thread (Ethicon LLC, Puerto Rico, USA). In order to mimic CDH damage ex vivo, part of the right side of the diaphragm was surgically removed. An equal sized and shaped 3% *w/v* (30 mg/mL) genipin cross-linked hydrogel was cut and manually sutured to the fresh diaphragm using 8-0 synthetic thread (Ethicon LLC, Puerto Rico, USA). Two different stimulation protocols were used to test the construct strength capability using a home-made stimulator. The first protocol requires a progressive increase of the strain rates, from 0 to 30% in 3 h, whereas the second one, to mimic the physiological movement of the diaphragm, requires a progressive increase of both frequency and strain rates (10, 30, 50 beat/min, with 10 or 30% of strain). Once stimulated, samples were collected, fixed in 4% PFA and analyzed.

### 2.14. Ex Vivo CDH Numerical Modeling

We developed a finite element (FE) model of a diaphragm repaired with a path according to its morphology. The general-purpose FE software ABAQUS Standard (SIMULIA^TM^, Dassault Systems^®^) was adopted for analysis. PDMS membrane, muscular tissue, central tendon, and hydrogel patch were modeled with hyperelastic constitutive models. The constitutive parameters of each model were obtained according to experimental stress–strain data. Since these materials show a quasi-incompressible mechanical behavior, the geometry was meshed with hybrid 3D solid elements. The interaction between the diaphragm and the PDMS membrane was modeled with contact surfaces, assuming a null friction. The external edges of the PDMS membrane were fixed to simulate the effective boundary conditions in the bioreactor. External edges of the diaphragm were fixed to the upper surface of the PDMS to simulate the suture fixation. A perfect bounding between hydrogel patch and muscle regions was considered. Nonlinear static analysis was developed to simulate the inflation of the PDMS membrane and the consequent deformation of the diaphragm under application of a uniform pressure to the lower surface of the membrane. The pressure was controlled considering the value of radial strain in the PDMS membrane in order to mimic the mechanical conditions imposed by the experiments.

### 2.15. Coating Experiments

Six-well plates were coated with 200 μL of neutralized dECM and maintained for 3 h at 37 °C, 5% CO_2_, 10% oxygen tension in a humidified chamber, so to allow the generation of the coated gel. After 3 washes in sterile PBS 1×, hSKMCs and hFbs were co-seeded at a ratio of 85–15%. Cells were maintained in proliferating medium for 4 days to reach confluence and then treated with fusion medium composed of αMEM (Gibco-Fischer Scientific, Milan, Italy) supplemented with 2% horse serum (Gibco-Fischer Scientific, Milan, Italy), 10 μg/mL insulin and 1% pen/strep for 3 more days. Myogenic index was calculated as the number of nuclei (greater than three) residing in myosin heavy chain (MHC)-positive fibers, divided by the number of total nuclei. Results were expressed as the number of cells per field.

### 2.16. In Vivo CDH

BALB/c Rag2^−/−^γc^−/−^ mice were used according to approved protocols (Protocols N. 1103/2016 and N. 418/2020-PR approved by animal wellness local ethics committee OPBA (Organismo per il Benessere Animale, Padova) and the Italian Ministry of Health). Animals were operated on under general anesthesia following the protocol described in Trevisan et al. [24]. Briefly, after a median superior incision, a 3 × 5 mm hole was surgically created in the left side of the diaphragm. Afterwards the defect was closed by gluing a 3% *w/v* (30 mg/mL) cross-linked hydrogel. Organs were then repositioned into the abdominal cavity; the abdominal wall was closed in two layers, and the animals left to recover under a heating lamp. Treated mice were euthanized by cervical dislocation at 3 (*n* = 3) and 7 (*n* = 3) days post-surgery.

### 2.17. Statistical Analysis

Data are expressed as mean ± s.e.m. or s.d. For immunofluorescence and histological analyses, at least 15 random high-power field areas were considered per analyzed muscle. Statistical significance was determined using a parametric Student’s *t* test after D’Agostino–Pearson normality test, or non-parametric Kruskal–Wallis test using GraphPad Prism 6v. A *p*-value below 0.05 was considered statistically significant.

## 3. Results

### 3.1. Porcine Diaphragmatic Decellularized ECM Characterization

**Figure 1:**
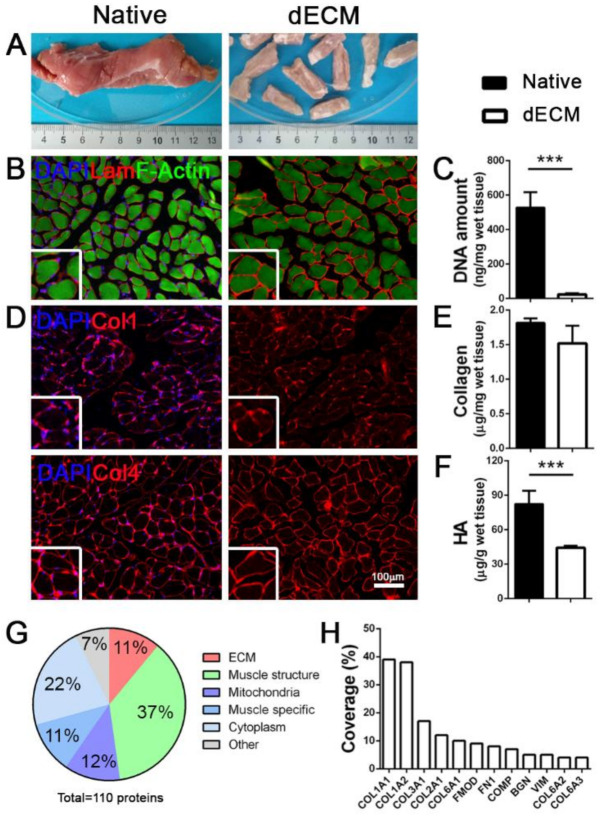
Porcine decellularized diaphragmatic muscle tissue characterization. (**A**) Gross appearance of native and decellularized (dECM) porcine tissue. (**B**) Representative immunofluorescence images of porcine diaphragm before and after decellularization; Laminin in red, F-Actin in green. (**C**) Quantification of DNA amount before and after decellularization. (**D)** Representative immunofluorescence images of collagens (collagen 1 and 4) in diaphragmatic muscle before and after decellularization. Nuclei are counterstained with DAPI (blue). (**E**,**F**) Quantification of total collagen and hyaluronic acid (HA) in native and decellularized porcine diaphragms. (**G**) Graphic representation of total proteins detected in decellularized samples. (**H**). Percentage of coverage of specific ECM proteins detected in decellularized samples. *** *p* < 0.001.

The starting material for the production of hydrogels was porcine diaphragmatic decellularized ECM (dECM). Small pieces of dissected diaphragm were treated according to the enzymatic decellularization protocol to ensure complete removal of the cellular component from the native tissue (Figure 1A).

We obtained an efficient decellularization, observing a complete cell removal in the treated samples (Figure 1B,C), together with the preservation of important ECM proteins. Notably, structural proteins such as laminin and collagens (Col1 and Col4) were maintained in the dECM, hence preserving both the architecture (Figure 1D) and the composition of the original diaphragmatic tissue; this was further confirmed by the total collagen quantification that resulted in similar values for both the native and decellularized samples (Figure 1E). Conversely, as previously reported [30], we observed a strong impact of the decellularization process on glycosaminoglycans, specifically on hyaluronic acid (HA) that was significantly decreased in respect to the native tissue (Figure 1F).

Of the 110 total discovered proteins (Table S1), proteomic analysis demonstrated that porcine decellularized diaphragms were composed of about 11% of ECM structural proteins, of which different isoforms of Col1 and Col6 constituted the most represented fraction (Figure 1G,H). As expected, the most abundant category comprised muscle contraction proteins (37%; Figure 1G) with identified different isoforms of myosin and actin (Figure S1A), but also small proteins related to skeletal muscle function (11%) and mitochondria metabolism (12%). Notably, we found an overlap of more than 80% of total detected proteins among all the analyzed samples (Figure S1B), suggesting this decellularization protocol had a good reproducibility.

### 3.2. Diaphragm dECM-Derived Hydrogel Preparation and Characterization

**Figure 2:**
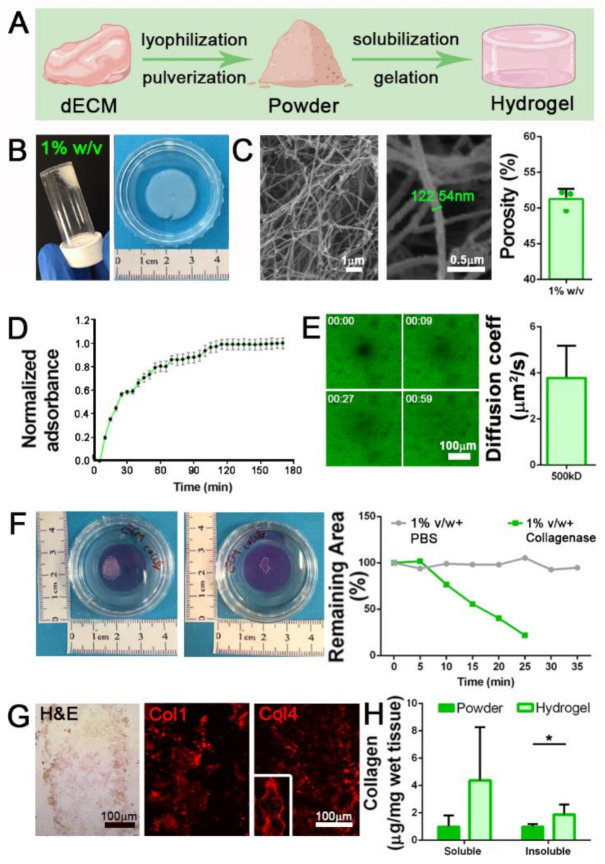
Diaphragmatic dECM-derived hydrogels production. (**A**) Graphical representation of the three-steps protocol to produce diaphragmatic dECM-derived hydrogels. (**B**) Gross appearance of 1% *w/v* jellified hydrogel. (**C**) SEM analysis of 1% *w/v* dECM-derived hydrogel and porosity calculation. (**D**) Turbidimetric gelation kinetics of 1% *w/v* dECM-derived hydrogel, reported in terms of normalized absorbance (±s.e.m.) vs. time. (**E**) Fluorescence recovery after photobleaching (FRAP) analysis of 1% *w/v* hydrogels. Diffusion coefficient for fluorescein isothiocyanate (FITC)-dextran of 500 kDa was calculated by fitting the FRAP data with the Uniform Disk Model (UDM). (**F**) Degradation assay of 1% *w/v* hydrogels using collagenase II. PBS: 1% *w/v* hydrogels incubated with PBS (no collagenase II). (**G**) Characterization of 1% *w/v* hydrogel with hematoxylin and eosin (H&E) stain, and immunofluorescence for collagen 1 (Col1) and collagen 4 (Col4). (**H**) Quantification of soluble and insoluble collagen before (powder) and after hydrogel production. *: *p* < 0.05.

To obtain batches of dECM-derived hydrogel, we followed a three-step protocol that exploits the rearrangement of collagen fibrils after acidic enzymatic digestion and the restoring of physiological conditions [11] (Figure 2A).

Decellularized tissue (dECM) was lyophilized, pulverized, and enzymatically subjected to pepsin digestion in acetic acid for 48 h. This time was required to obtain a milky and homogeneous solution, which after restoration of physiological osmolarity and pH values (7.4) can be triggered to jellify under controlled temperature conditions (37 °C). We successfully jellified different volumes of neutralized dECM (from 50 μL to 3 mL) and prepared hydrogels with different powder concentrations (10, 20, 30 mg/mL). At first, 1% *w/v* (10 mg/mL) hydrogels were prepared and analyzed (Figure 2B). Ultrastructural analysis demonstrated that the gelation process was able to induce the formation of a strictly organized network composed of filaments with size comparable to collagen fibrils (Figure 2C) and a porosity compatible with cell survival and migration inside the scaffold (51.2 ± 1.4%; Figure 2C).

The turbidimetric gelation kinetics were analyzed following published protocol [27]. Although the gelation time was not always identical in all the analyzed samples, we calculated a mean gelation half time ($t_{1/2}$) of 26.1 min, together with the gelation rate *S* that was evaluated as 0.013, and the lag time ($t_{lag}$) corresponding to 10 min and 30 s (Figure 2D). Diffusion in hydrogels was studied using the fluorescence recovery after photobleaching (FRAP) technique [31]. In agreement with published protocols [32], we measured the recovery kinetics of fluorescein-labeled dextran (500 kDa) after photobleaching light pulse in 1% w/v hydrogels, demonstrating that they possessed a mesh size that allows the diffusion of molecules with a diffusion coefficient of 3.8 ± 1.3 μm^2^/s (Figure 2E).

ECM remodeling represents a mandatory process for biocompatible hydrogel production. Hydrogel degradation should be achieved to favor cell deposition of newly ECM proteins, which act as a more specific assembled microenvironment. To investigate the dECM-derived hydrogel degradation susceptibility, we set up an in vitro assay exploiting the ability of the enzyme collagenase II, active in the native SKM microenvironment for the physiological turnover of ECM proteins, to cleave the triple helical protein chains of our samples. Interestingly, 1% *w/v* hydrogels were progressively digested by the action of the enzyme, as highlighted by the reduction of the hydrogel area until complete dissolution after 25 min of treatment, in comparison with hydrogels that were instead treated with PBS control solution (Figure 2F).

As expected after proteomic analysis of dECM powder, we were able to obtain well-structured hydrogels via re-formation of collagen fibrils as demonstrated by the intense expression of Col1 and Col4 in hydrogel sections (Figure 2G). Through quantification of the total collagen amount in both formulations, we discovered a substantial increase in both soluble and insoluble collagen content in hydrogel compared to the powder, with a more significant increment of insoluble collagen (Figure 2H).

All these results suggested that 1% *w/v* hydrogels can be obtained starting from diaphragmatic dECM, and the characteristics they possess in terms of ultrastructure and composition indicate a potential role as SKM mimicking scaffolds.

### 3.3. Diaphragm dECM-Derived Hydrogel as Stand-Alone Acellular Patches for Application in SKM Malformations

**Figure 3:**
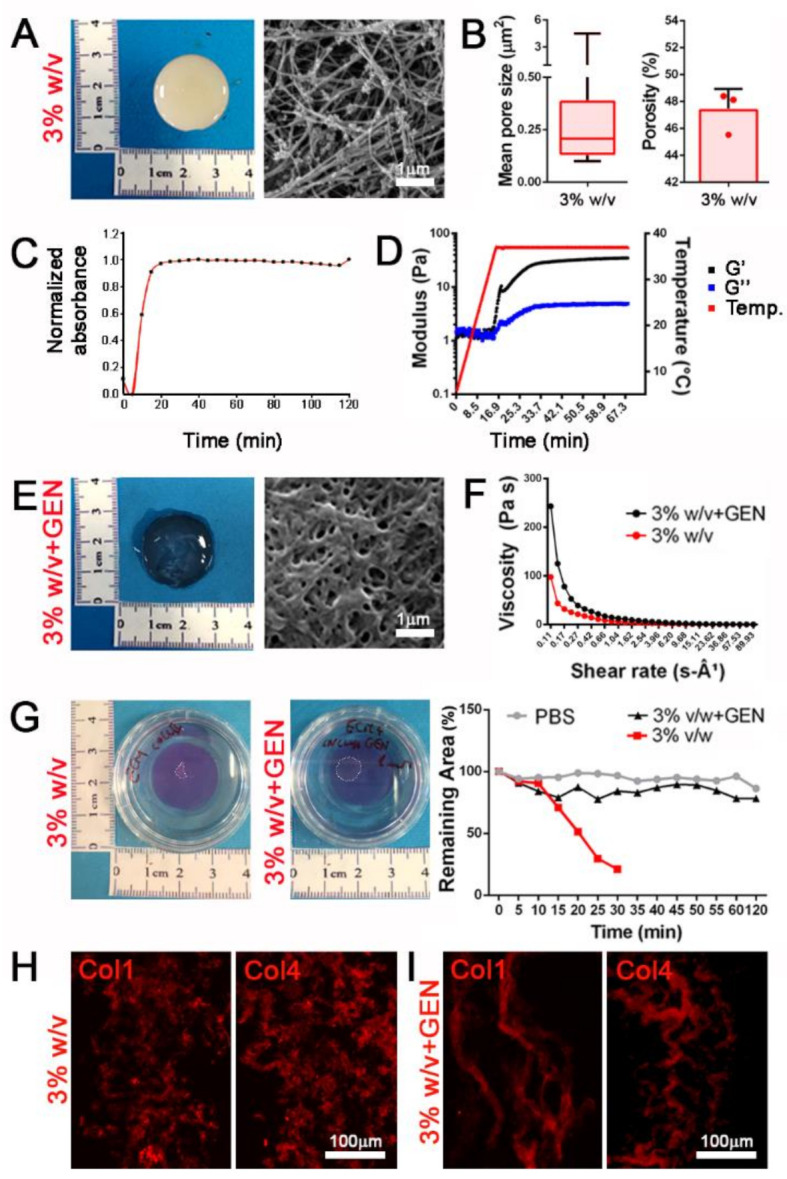
dECM-derived hydrogels can be chemically modified to increase the mechanical properties. (**A**) Gross appearance and ultrastructure of 3% *w/v* hydrogels. (**B**) Mean pore size and porosity of 3% *w/v* hydrogels. (**C**) Turbidimetric gelation kinetics of 3% *v/w* dECM-derived hydrogel, reported in terms of normalized absorbance vs. time. (**D**) Storage (G′) and loss (G″) moduli of 3% *w/v* dECM-derived hydrogel obtained from rheological analysis increasing temperature (from 0 to 40 °C) during time (from 0 to 60 min). (**E**) Gross appearance and ultrastructure of 3% *w/v* hydrogels after crosslinking with genipin (+GEN). (**F**) Viscosity properties under different shear rate of 3% *w/v* hydrogels with and without genipin crosslinking. (**G**) Degradation assay of 3% *w/v* hydrogels with and without genipin crosslinking using collagenase II. PBS: 3% *w/v* hydrogels without genipin and incubated with PBS (no collagenase II). (**H**,**I**) Immunofluorescence staining for the detection of Col1 and Col4 in 3% *w/v* hydrogels and cross-linked hydrogels with 1 mM genipin (3% *w/v* + GEN).

With a special focus on potential clinical applications of these hydrogels as stand-alone patches for SKM malformations, we moved to improve the mechanical stability in terms of viscoelastic properties and degradation rate. We observed that 20 and 30 mg/mL hydrogels (2 and 3% w/v, respectively) possessed a more compact outer structure (Figure 3A and Figure S2A) and the increment of initial concentrations of ECM powder impacted also in the inner arrangement, with a more intricated network of fibrils and smaller pores in respect to 1% w/v hydrogels (Figure 3B and Figure S2B).

These compositions were also characterized by a more reproducible and faster turbidimetric kinetic (Figure 3C). Rheological analysis in an oscillatory temperature ramp demonstrated that gel-like properties were observable when increasing temperature, with a higher and stabilized storage modulus (G′) than the loss modulus (G″) when hydrogels were brought up to a temperature of 37 °C (Figure 3D).

Chemical crosslinking represents a useful method to obtain stable hydrogels and reach suitable mechanical properties [33]. Taking advantage of the abundant presence of collagen, we cross-linked our hydrogels using genipin, a natural crosslinker molecule that can bridge free amino groups of lysine or hydroxylysine residues of different polypeptide chains by monomeric or oligomeric crosslinks in collagen [34]. Cross-linked hydrogels presented a more densely packed inner architecture (Figure 3E and Figure S2C) and collagen packaging (Figure 3H,I) resulting in a 2-fold significant increase of viscosity as opposed to the same concentration of non-cross-linked hydrogels, and demonstrating a more suitable behavior when subjected to shear stress (Figure 3F and Figure S2D). Moreover, cross-linked hydrogels appeared to be unaffected by enzymatic degradation, in contrast to non-cross-linked samples, suggesting a potential resistance when used as in vivo tissue substitutes (Figure 3G and Figure S2E).

These results highlighted a strong tunability of dECM-derived hydrogel properties, which could be treated to increase the mechanical stability that is needed for tissue replacement purposes in more complex and stressing models, such as in an in vivo environment.

### 3.4. Diaphragm dECM-Derived Hydrogel as Acellular Patch in a Mechanically Stimulated CDH Ex Vivo Model

**Figure 4:**
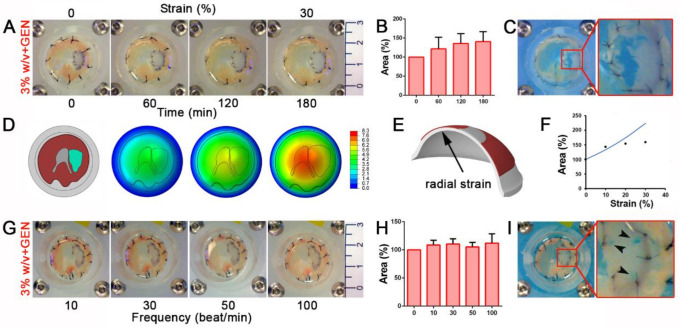
Cross-linked diaphragm dECM-derived hydrogel patches resist both continuous and frequent mechanical stimuli in a diaphragmatic hernia (CDH) ex vivo model. (**A**) Gross appearance of 3% *w/v* cross-linked hydrogel patches sutured on the defective diaphragms and mechanically stimulated from 0 to 30% strain for 3 h. (**B**) Patch area at each point during incremental strain stimulation. Percentage was calculated respect to the initial area of non-stimulated patch. (**C**) Gross appearance of patches encountering breakage events when strain was at maximal level (60%). (**D**) Finite element (FE) model of mouse diaphragm with dECM-derived patch fixed to polydimethylsiloxane (PDMS) membrane and magnitude displacement fields at different levels of PDMS membrane radial strain; the patch is depicted in cyan. (**E**) Detail of the region of PDMS membrane in which radial strain is evaluated. (**F**) Comparison between experimental data and numerical evaluation of patch area, projected on the horizontal plane. Percentage was calculated with respect to the initial area of non-stimulated patch. (**G**) Gross appearance of mechanically stimulated cross-linked patches subjected to increasing frequency (10, 30, 50, 100 beat/min with a 10% strain applied). (**H**) Patch area at each tested frequency. Percentage was calculated as in the previous cases. (**I**) Gross appearance of breaking points (black arrow heads) occurring to the patch around the stitches when strain was increased at 30% and frequency of 100 beat/min.

Having successfully achieved an improvement with the crosslinking process, we firstly tested 3% *w/v* cross-linked hydrogels in a CDH ex vivo mouse model. After diaphragm explant, we induced the formation of a small hole (about 4 mm diameter) to reproduce a defect similar to in vivo CDH (Figure S3A). Cross-linked hydrogels were sutured above the defect, completely resolving the surgical damage. When repaired, diaphragms were subjected to a slow and incremental radial mechanical strain from 0 to 30% in a home-made bioreactor specifically designed for the purpose. The diaphragm was firmly placed on the upper surface of a polydimethylsiloxane (PDMS) circular membrane that was bounded with its external edge. A hydraulic circuit was adopted to apply pressure on the lower surface of the PDMS membrane, thus inducing its inflation and the consequent stretching of the diaphragm. In these conditions, hydrogels demonstrated a high resistant behavior without evident signs of breaking (Figure 4A) until maximal deformation was employed.

The cross-linked patch was able to adapt to the increasing strain applied to the diaphragm by proportionally expanding its area (Figure 4B and Figure S3B). A large breakage occurred solely when extreme strain (60%), far exceeding the physiological condition, was applied (Figure 4C).

To better evaluate the deformation of the repaired diaphragm, we developed a Finite Element (FE) model to simulate the interaction between the diaphragm and the PDMS membrane (Figure 4D). Assuming an elastic behavior and a perfect bounding between the patch and the surrounding tissue (Figure S3C), we evaluated the change of the patch area as a function of the level of radial strain in the PDMS membrane (Figure 4E) and finally we compared the corresponding curve of the model with experimental data. Results showed a similar behavior between the model and experiments up to 10% of radial strain, whereas above this value the responses differed considerably. The numerical model showed an increase of the deformed patch area, whereas a tendency to a constant value was noticed in the experimental data (Figure 4F). We assumed that this difference was due to a lack of integrity of the bounds between patch and surrounding tissue.

To finely mimic in vivo physiological diaphragmatic movements, we applied a strain protocol using a progressively increasing frequency of mechanical stimuli (starting from 10 to 100 beats every minute [beat/min]) with a constant physiological 10% of strain (Figure 4G and Movie S1). Cross-linked patches underwent an initial adaptation when subjected to 10 and 30 beat/min, but remained constantly stretched when the number of beats increased (Figure 4H). Only when strain and frequency parameters were forced to reach extreme physiological values (30% of strain and 100 beat/min) did hydrogel patches begun to show breaking points around the stitches used to sew the sample to the muscle (Figure 4I).

Considering the overall mechanical behavior of the patch and the level of strain that is normally applied to promote muscle fiber formation and contraction (under 10%), experimental data demonstrated a potential role of our dECM-derived hydrogel as a stand-alone patch in a CDH in vivo model.

### 3.5. Diaphragm dECM-Derived Hydrogel as Suitable Material for In Vivo Applications

Before testing hydrogel application in an in vivo setting, we verified the in vitro biocompatibility and relative effect on myogenic cell behavior. We set up in vitro experiments in which dECM-derived hydrogels were used as plate coating for 2D cultures of a mixed human SKM cell (hSKMC) and human fibroblast (hFb) population (85–15% ratio) in order to investigate the hydrogel influence on seeded cells. Of note, the choice of combining these two cell types was dictated by the idea of using the majority of the population that in vivo would eventually colonize and replace the implant. Interestingly, seeded cells were able to actively proliferate, as indicated by the marker Ki67 strongly increasing in number during the first four days of culture (382.3 ± 133.1 cells/field at day 2; 499.0 ± 113.1 cells/field at day 4; Figure S4A). Moreover, when the cells were induced to differentiate, hydrogel coating was able to support cell fusion and myotubes’ formation, as highlighted by the expression of myosin heavy chain (MHC) and the calculation of myogenic index (17.3 ± 7.2%; Figure S4B).

**Figure 5:**
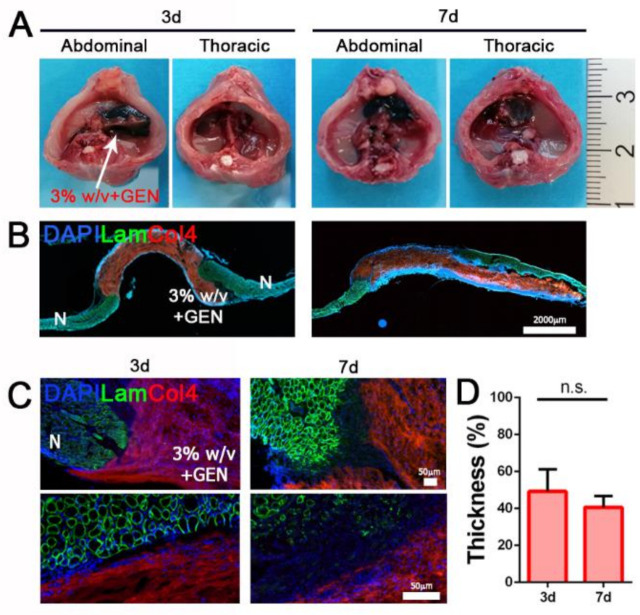
Diaphragm dECM-derived hydrogel in vivo implantation. (**A**) Gross appearance of abdominal and thoracic sides of treated diaphragms after 3 and 7 days. (**B**) Representative images of transversal section of treated diaphragms after 3 and 7 days. (**C**) Immunofluorescence staining for the detection of Laminin and Col4. (**D**) Thickness of 3% w/v + GEN hydrogel after in vivo implantation expressed as percentage of the initial thickness (100%). N: native muscle. n.s.: non-significant.

For in vivo experiments, we implanted 3% *w/v* cross-linked (3% *w/v* + GEN) hydrogels in a well-established surgical model of CDH [24] in Rag2^−/−^γc^−/−^ mice, in order to investigate the ability of this biomaterial to withstand to physiological strain and integrate with the recipient muscle. Treated diaphragms were analyzed three and seven days after implantation without detecting liver herniation, demonstrating a good strength capability of implanted hydrogels (Figure 5A).

Moreover, since day 3 post implantation, hydrogel patches were strongly repopulated by resident cells, confirming in vivo biocompatible features that we previously detected in vitro. As expected, diaphragm dECM-derived hydrogels were partially degraded and reabsorbed during the entire period of treatment, losing just over 50% of pre-implantation thickness (49.2 ± 11.7% after three days; 40.6 ± 6.0% after seven days), but evidently without affecting their mechanical strength.

Taken together, these preliminary in vivo data confirmed that diaphragm dECM-derived hydrogels are a biocompatible material with appropriate mechanical behavior and therefore suitable as stand-alone patches for diaphragm applications.

## 4. Discussion

Decellularized SKM ECM appears to be an inimitable environment for muscle cells thanks to the preservation of native architecture and composition, and the presence of embedded growth factors released by resident cells before undergoing decellularization [30,35]. Nowadays, this type of scaffold represents the best compromise between physiologic environment and synthetic materials when used as a substitute in SKM defects. Building upon previous findings in our research group on murine diaphragmatic dECM [24,36], we moved to produce a porcine diaphragmatic dECM to adopt a smart scaled-up approach and potentially manufacture different scaffold types. We adapted the detergent-enzymatic decellularization process used for murine samples to porcine muscle, setting up a reproducible technique to obtain a completely acellular scaffold that resembles native diaphragmatic architecture and protein composition.

Among the potential roles and applications of this tissue-specific bioscaffold devoid of cells, we chose to develop batch-to-batch reproducible formulations of dECM-derived hydrogels with the aim of producing a new highly versatile biomaterial that closely resembles the native diaphragmatic ECM composition. Production of hydrogels was possible by exploiting the presence of collagen proteins in lyophilized and pulverized porcine dECM, which were preserved following the decellularization process, as confirmed by the mass spectrometry analysis. Notably, through proteomic analysis, we also verified the maintenance of proteins of the acto-myosin apparatus and its auxiliary components, as well as the Ca^2+^-handling protein complex involved in the regulation of excitation–contraction coupling. These detected proteins are specific and determinant components of SKM, including diaphragm [37], strongly supporting the efficacy of the detergent-enzymatic decellularization protocol we adopted in terms of maintenance of the native tissue composition. As performed with other muscle types [11,38,39,40], we followed a well-established jellification protocol for hydrogel production, starting from this tissue-specific material, digesting powders and inducing the re-formation of collagen fibrils [41]. In this way we obtained diaphragmatic dECM-derived hydrogels with interesting ultrastructure characteristics and mechanical stability, which conferred on them several potential applications. The gelation process triggered the formation of an intricate inner ultrastructure, with the generation of a porous scaffold able to permit nutrients and gas diffusion, especially in the less concentrated formulation (1% w/v). Hydrogels possessed texture, which gave them the capability to be easily manipulated while maintaining a compact shape. Moreover, we observed another important behavior in support of the possibility of implementing these hydrogels as a cellular environment for both in vitro and in vivo settings: the susceptibility to degradation when subjected to enzymatic digestion. This feature endorses the application of dECM-derived hydrogels as biocompatible cellular scaffolds, because blended or host-derived cells can possibly start to produce their own ECM while hydrogel enzymatic degradation occurs, giving rise to a cell specific and peculiar microenvironment. We demonstrated a tunability for these hydrogels in terms of composition, because by varying the initial ECM powder concentrations we were able to induce different changes in the ultrastructural architecture and mechanical strength. This tunability represents a relevant characteristic, making possible the generation of bioactive and resistant acellular constructs. Once we demonstrated that the gelation properties of our hydrogels were comparable to other developed dECM-derived hydrogels [5,11,42], we investigated the foreseen biological properties at multiple levels. We confirmed the presence of distinctive SKM ECM-related proteins in our hydrogels, such as collagens. Interestingly, after detecting and quantifying soluble and insoluble collagen, we observed that in the analyzed hydrogel formulation there was an increased content of both collagen forms. Although an indirect approach, analysis of collagen solubility has been frequently adopted to measure the relative amounts of protein crosslinking levels in various types of tissue [43,44]. Hydrogels should possess specific mechanical characteristics, including viscoelastic properties, when designed as a possible biological application for in vivo muscle defect repair [45]. Multiple extrudable hydrogels have been used to replenish large muscle defects, such as volumetric muscle loss [46,47,48]. The nature of these defects allows for hydrogel application as a filling material, whereas other SKM malformations such as CDH are not amendable with this type of approach, due to the complete loss of a muscle portion that cannot be filled with a jelling solution. Different biologic patches were used and clinically tested for the treatment of CDH. Unfortunately, none of the tested options gave strong amelioration compared to the application of the well-known and largely used synthetic material [20,21]. Failure of these strategies probably lies in two intimately linked aspects: the chosen biologic materials derived from organs or tissues different from SKM, on one hand, did not allow the necessary mechanical strength for incessant diaphragm movements, and, on the other, did not supply important specific factors and proteins to recipient cells. We demonstrated, indeed, that the use of diaphragm-derived patches for the treatment of surgically induced CDH in mice can overcome the limits showed by the application of other biologic or synthetic materials, eliminating hernia recurrence and improving survival and diaphragm function recovery [24]. From the biological point of view, hydrogels developed in this work already represent a tissue-specific tool for the treatment of CDH. The mechanical properties, instead, can be tuned to increase their strength when subjected to external mechanical stimuli mimicking those found in diaphragmatic in vivo condition. Several studies utilized the natural crosslinker genipin for increasing bio-stability and mechanical strength of dECM-derived hydrogels when exploited as potential clinical scaffolds in in vivo models [49,50,51]. We demonstrated that the mechanism of action of genipin worked optimally when applied to the more concentrated hydrogels we produced, inducing a desired increase of mechanical properties and a long-term resistance to collagenase degradation in comparison with non-cross-linked hydrogels. We tested a new application with potential clinical implications, reproducing at first in vitro diaphragmatic defects mimicking the physiological movement of the muscle that we repaired using cross-linked dECM-derived hydrogels as acellular patches. Hydrogels demonstrated a suitable strength to mechanical stress, when applying either an incremental or a high frequency cyclical stimulus to the diaphragm at physiological or even higher strain levels. We tested these properties also through in silico analyses that were aimed at evaluating the mechanical response of diaphragm-patch compound [52]. Then, cross-linked hydrogels were used in vivo as stand-alone patches for the closure of surgically induced CDH, confirming in a complex biologic environment their ability to withstand repeated mechanical strain due to physiological and spontaneous breathing. Moreover, as shown in other fields [5,53], we can consider our hydrogels to constitute a biocompatible and enriched muscle environment, since we observed from the first days after implantation a strong hydrogel repopulation by recipient cells. These good results confirmed that our compact cross-linked hydrogel could be potentially utilized in a clinical setting for the treatment of CDH, thus constituting a new easy-to-produce tissue-specific biological patch able to close the defect occurring in the diaphragm. Certainly, further in vivo experiments are required, in both small and large animals, performing longer time point analyses and investigating implant functionality, in order to evaluate hydrogel degradation rate or substitution by new muscle tissue regeneration. The proposed application envisages an innovative approach for the treatment of CDH since suitable sized and batch-to-batch identical scaffolds may be produced and applied to repair diaphragmatic defects, overcoming the limits related to donor availability in clinical practice.

## 5. Conclusions

In this study we developed a new product starting from diaphragmatic porcine dECM that can be transformed into tunable hydrogels with specific characteristics in terms of mechanical properties and biocompatibility. We propose novel reinforced hydrogels that can be used as acellular biological patches in the treatment of SKM defects, especially diaphragmatic malformations such as CDH, by exploiting their capacity to resist mechanical stresses. In a future scenario, in addition to the need for further in vitro hydrogel validation experiments in more complex systems and in vivo treatments for longer times before arriving at a possible clinical translation, we should also optimize the production of hydrogels to obtain an even more batch-to-batch reproducible product, overcoming the limitations related to source variability, for example, pooling together different diaphragm samples to obtain a more standardized product, or blending cells inside the hydrogel to generate implantable living muscle patches.

Finally, considering the great potential that recent work has shown for the use of 3D printing of biological materials for on-demand organ production with transplant purposes [54,55,56], bio-printability of our dECM-derived hydrogels should be investigated in the future, since bioprinting is now considered the next-generation technology to produce more complex and reliable 3D SKM tissues.

## Data Availability

The data presented in this study are available on request from the corresponding author.

## Supplementary Materials

The following are available online at https://www.mdpi.com/article/10.3390/biomedicines9070709/s1, Figure S1: Muscle-specific proteins in decellularized samples, Figure S2: 2% *w/v* dECM-derived hydrogel characterization, Figure S3: Preparation and analysis of cross-linked 3% *w/v* dECM-derived hydrogel application in ex vivo CDH model, Figure S4: In vitro hydrogel biocompatibility. Table S1: List of proteins found in dECM with mass spectrometry, Table S2: List of primary and secondary antibodies used. Movie S1: Cross-linked 3% *w/v* hydrogel mechanical stimulation in ex vivo CDH model.

## References

[1] Dzobo K., Thomford N.E., Senthebane D.A., Shipanga H., Rowe A., Dandara C., Pillay M., Motaung K.S.C.M. (2018). Advances in regenerative medicine and tissue engineering: Innovation and transformation of medicine. Stem Cells Int..

[2] Simon L.T.R., Masters K.S. (2020). Disease-Inspired Tissue Engineering: Investigation of Cardiovascular Pathologies. ACS Biomater. Sci. Eng..

[3] Kanetaka K., Kobayashi S., Eguchi S. (2018). Regenerative medicine for the esophagus. Surg. Today.

[4] Lim W.L., Liau L.L., Ng M.H., Chowdhury S.R., Law J.X. (2019). Current Progress in Tendon and Ligament Tissue Engineering. Tissue Eng. Regen. Med..

[5] Giobbe G.G., Crowley C., Luni C., Campinoti S., Khedr M., Kretzschmar K., De Santis M.M., Zambaiti E., Michielin F., Meran L. (2019). Extracellular matrix hydrogel derived from decellularized tissues enables endodermal organoid culture. Nat. Commun..

[6] Luo X., Fong E.L.S., Zhu C., Lin Q.X.X., Xiong M., Li A., Li T., Benoukraf T., Yu H., Liu S. (2020). Hydrogel-Based Colorectal Cancer Organoid Co-Culture Models. Acta Biomater..

[7] Xu Y., Zhou J., Liu C., Zhang S., Gao F., Guo W., Sun X., Zhang C., Li H., Rao Z. (2021). Understanding the role of tissue-specific decellularized spinal cord matrix hydrogel for neural stem/progenitor cell microenvironment reconstruction and spinal cord injury. Biomaterials.

[8] Fernandes-Cunha G.M., Chen K.M., Chen F., Le P., Han J.H., Mahajan L.A., Lee H.J., Na K.S., Myung D. (2020). In situ-forming collagen hydrogel crosslinked via multi-functional PEG as a matrix therapy for corneal defects. Sci. Rep..

[9] Boso D., Maghin E., Carraro E., Giagante M., Pavan P., Piccoli M. (2020). Extracellular matrix-derived hydrogels as biomaterial for different skeletal muscle tissue replacements. Materials.

[10] Brightman A.O., Rajwa B.P., Sturgis J.E., McCallister M.E., Robinson J.P., Voytik-Harbin S.L. (2000). Time-lapse confocal reflection microscopy of collagen fibrillogenesis and extracellular matrix assembly in vitro. Biopolymers.

[11] Choi Y.J., Jun Y.J., Kim D.Y., Yi H.G., Chae S.H., Kang J., Lee J., Gao G., Kong J.S., Jang J. (2019). A 3D cell printed muscle construct with tissue-derived bioink for the treatment of volumetric muscle loss. Biomaterials.

[12] Kim J.H., Kim I., Seol Y.J., Ko I.K., Yoo J.J., Atala A., Lee S.J. (2020). Neural cell integration into 3D bioprinted skeletal muscle constructs accelerates restoration of muscle function. Nat. Commun..

[13] Rao N., Agmon G., Tierney M.T., Ungerleider J.L., Braden R.L., Sacco A., Christman K.L. (2017). Engineering an Injectable Muscle-Specific Microenvironment for Improved Cell Delivery Using a Nanofibrous Extracellular Matrix Hydrogel. ACS Nano.

[14] Annabi N., Nichol J.W., Zhong X., Ji C., Koshy S., Khademhosseini A., Dehghani F. (2010). Controlling the porosity and microarchitecture of hydrogels for tissue engineering. Tissue Eng. Part B Rev..

[15] Urciuolo A., Poli I., Brandolino L., Raffa P., Scattolini V., Laterza C., Giobbe G.G., Zambaiti E., Selmin G., Magnussen M. (2020). Intravital three-dimensional bioprinting. Nat. Biomed. Eng..

[16] Hu W., Wang Z., Xiao Y., Zhang S., Wang J. (2019). Advances in crosslinking strategies of biomedical hydrogels. Biomater. Sci..

[17] De Kort L.M.O., Bax K.N.M.A. (1996). Prosthetic Patches Used to Close Congenital Diaphragmatic Defects Behave Well: A Long-Term Follow-Up Study. Proc. Eur. J. Pediatric Surg. Georg Thieme Verl..

[18] Laituri C.A., Garey C.L., Valusek P.A., Fike F.B., Kaye A.J., Ostlie D.J., Snyder C.L., St. Peter S.D.S. (2010). Outcome of congenital diaphragmatic hernia repair depending on patch type. Eur. J. Pediatr. Surg..

[19] Moss R.L., Chen C.M., Harrison M.R. (2001). Prosthetic patch durability in congenital diaphragmatic hernia: A long-term follow-up study. J. Pediatr. Surg..

[20] Grethel E.J., Cortes R.A., Wagner A.J., Clifton M.S., Lee H., Farmer D.L., Harrison M.R., Keller R.L., Nobuhara K.K. (2006). Prosthetic patches for congenital diaphragmatic hernia repair: Surgisis vs Gore-Tex. J. Pediatr. Surg..

[21] Romao R.L.P., Nasr A., Chiu P.P.L., Langer J.C. (2012). What is the best prosthetic material for patch repair of congenital diaphragmatic hernia? Comparison and meta-analysis of porcine small intestinal submucosa and polytetrafluoroethylene. J. Pediatr. Surg..

[22] Pickering M., Jones J.F.X. (2002). The diaphragm: Two physiological muscles in one. J. Anat..

[23] Fogarty M.J., Sieck G.C. (2019). Evolution and functional differentiation of the diaphragm muscle of mammals. Compr. Physiol..

[24] Trevisan C., Maghin E., Dedja A., Caccin P., de Cesare N., Franzin C., Boso D., Pesce P., Caicci F., Boldrin F. (2019). Allogenic tissue-specific decellularized scaffolds promote long-term muscle innervation and functional recovery in a surgical diaphragmatic hernia model. Acta Biomater..

[25] Hynes R.O., Naba A. (2012). Overview of the matrisome-An inventory of extracellular matrix constituents and functions. Cold Spring Harb. Perspect. Biol..

[26] Braeckmans K., Peeters L., Sanders N.N., De Smedt S.C., Demeester J. (2003). Three-dimensional fluorescence recovery after photobleaching with the confocal scanning laser microscope. Biophys. J..

[27] Freytes D.O., Martin J., Velankar S.S., Lee A.S., Badylak S.F. (2008). Preparation and rheological characterization of a gel form of the porcine urinary bladder matrix. Biomaterials.

[28] Fercana G.R., Yerneni S., Billaud M., Hill J.C., Van Ryzin P., Richards T.D., Sicari B.M., Johnson S.A., Badylak S.F., Campbell P.G. (2017). Perivascular extracellular matrix hydrogels mimic native matrix microarchitecture and promote angiogenesis via basic fibroblast growth factor. Biomaterials.

[29] Wolf M.T., Daly K.A., Brennan-Pierce E.P., Johnson S.A., Carruthers C.A., D’Amore A., Nagarkar S.P., Velankar S.S., Badylak S.F. (2012). A hydrogel derived from decellularized dermal extracellular matrix. Biomaterials.

[30] Piccoli M., Urbani L., Alvarez-Fallas M.E., Franzin C., Dedja A., Bertin E., Zuccolotto G., Rosato A., Pavan P., Elvassore N. (2016). Improvement of diaphragmatic performance through orthotopic application of decellularized extracellular matrix patch. Biomaterials.

[31] Vigata M., Meinert C., Hutmacher D.W., Bock N. (2020). Hydrogels as drug delivery systems: A review of current characterization and evaluation techniques. Pharmaceutics.

[32] Giobbe G.G., Zagallo M., Riello M., Serena E., Masi G., Barzon L., Di Camillo B., Elvassore N. (2012). Confined 3D microenvironment regulates early differentiation in human pluripotent stem cells. Biotechnol. Bioeng..

[33] Claudio-Rizo J.A., Delgado J., Quintero-Ortega I.A., Mata-Mata J.L., Mendoza-Novelo B. (2018). Decellularized ECM-Derived Hydrogels: Modification and Properties. Hydrogels.

[34] Sung H.W., Chang W.H., Ma C.Y., Lee M.H. (2003). Crosslinking of biological tissues using genipin and/or carbodiimide. J. Biomed. Mater. Res. Part A.

[35] Wolf M.T., Daly K.A., Reing J.E., Badylak S.F. (2012). Biologic scaffold composed of skeletal muscle extracellular matrix. Biomaterials.

[36] Trevisan C., Fallas M.E.A., Maghin E., Franzin C., Pavan P., Caccin P., Chiavegato A., Carraro E., Boso D., Boldrin F. (2019). Generation of a Functioning and Self-Renewing Diaphragmatic Muscle Construct. Stem Cells Transl. Med..

[37] Murphy M.M., Lawson J.A., Mathew S.J., Hutcheson D.A., Kardon G. (2011). Satellite cells, connective tissue fibroblasts and their interactions are crucial for muscle regeneration. Development.

[38] Das S., Kim S.W., Choi Y.J., Lee S., Lee S.H., Kong J.S., Park H.J., Cho D.W., Jang J. (2019). Decellularized extracellular matrix bioinks and the external stimuli to enhance cardiac tissue development in vitro. Acta Biomater..

[39] Fu Y., Fan X., Tian C., Luo J., Zhang Y., Deng L., Qin T., Lv Q. (2016). Decellularization of porcine skeletal muscle extracellular matrix for the formulation of a matrix hydrogel: A preliminary study. J. Cell. Mol. Med..

[40] Zhang D., Tan Q.W., Luo J.C., Lv Q. (2018). Evaluating the angiogenic potential of a novel temperature-sensitive gel scaffold derived from porcine skeletal muscle tissue. Biomed. Mater..

[41] Voytik-Harbin S.L., Brightman A.O., Waisner B.Z., Robinson J.P., Lamar C.H. (1998). Small intestinal submucosa: A tissue-derived extracellular matrix that promotes tissue-specific growth and differentiation of cells in vitro. Tissue Eng..

[42] Kang H.W., Lee S.J., Ko I.K., Kengla C., Yoo J.J., Atala A. (2016). A 3D bioprinting system to produce human-scale tissue constructs with structural integrity. Nat. Biotechnol..

[43] Badenhorst D., Maseko M., Tsotetsi O.J., Naidoo A., Brooksbank R., Norton G.R., Woodiwiss A.J. (2003). Cross-linking influences the impact of quantitative changes in myocardial collagen on cardiac stiffness and remodelling in hypertension in rats. Cardiovasc. Res..

[44] Reddy G.K. (2004). Cross-linking in collagen by nonenzymatic glycation increases the matrix stiffness in rabbit Achilles tendon. Exp. Diabesity Res..

[45] Lev R., Seliktar D. (2018). Hydrogel biomaterials and their therapeutic potential for muscle injuries and muscular dystrophies. J. R. Soc. Interface.

[46] Fischer M., Rikeit P., Knaus P., Coirault C. (2016). YAP-mediated mechanotransduction in skeletal muscle. Front. Physiol..

[47] Wu J., Matthias N., Bhalla S., Darabi R. (2021). Evaluation of the Therapeutic Potential of Human iPSCs in a Murine Model of VML. Mol. Ther..

[48] Rossi C.A., Flaibani M., Blaauw B., Pozzobon M., Figallo E., Reggiani C., Vitiello L., Elvassore N., De Coppi P. (2011). In vivo tissue engineering of functional skeletal muscle by freshly isolated satellite cells embedded in a photopolymerizable hydrogel. FASEB J..

[49] Výborný K., Vallová J., Kočí Z., Kekulová K., Jiráková K., Jendelová P., Hodan J., Kubinová Š. (2019). Genipin and EDC crosslinking of extracellular matrix hydrogel derived from human umbilical cord for neural tissue repair. Sci. Rep..

[50] Peng Y., Huang D., Li J., Liu S., Qing X., Shao Z. (2020). Genipin-crosslinked decellularized annulus fibrosus hydrogels induces tissue-specific differentiation of bone mesenchymal stem cells and intervertebral disc regeneration. J. Tissue Eng. Regen. Med..

[51] Nyambat B., Manga Y.B., Chen C.H., Gankhuyag U., Andi Pratomo W.P., Satapathy M.K., Chuang E.Y. (2020). New insight into natural extracellular matrix: Genipin cross-linked adipose-derived stem cell extracellular matrix gel for tissue engineering. Int. J. Mol. Sci..

[52] de Cesare N., Trevisan C., Maghin E., Piccoli M., Pavan P.G. (2018). A finite element analysis of diaphragmatic hernia repair on an animal model. J. Mech. Behav. Biomed. Mater..

[53] Sylvester C.B., Pugazenthi A., Grande-Allen K.J. (2021). Cell-Laden Bioactive Poly(ethylene glycol) Hydrogels for Studying Mesenchymal Stem Cell Behavior in Myocardial Infarct-Stiffness Microenvironments. Cardiovasc. Eng. Technol..

[54] Costantini M., Testa S., Mozetic P., Barbetta A., Fuoco C., Fornetti E., Tamiro F., Bernardini S., Jaroszewicz J., Święszkowski W. (2017). Microfluidic-enhanced 3D bioprinting of aligned myoblast-laden hydrogels leads to functionally organized myofibers in vitro and in vivo. Biomaterials.

[55] Kim J.H., Seol Y.-J., Ko I.K., Kang H.-W., Lee Y.K., Yoo J.J., Atala A., Lee S.J. (2018). 3D Bioprinted Human Skeletal Muscle Constructs for Muscle Function Restoration. Sci. Rep..

[56] Zhuang P., An J., Chua C.K., Tan L.P. (2020). Bioprinting of 3D in vitro skeletal muscle models: A review. Mater. Des..

