# Supplementary material for: Porcine Decellularized Diaphragm Hydrogel: A New Option for Skeletal Muscle Malformations
Source: Biomedicines. 2021 Jun 22;9(7):709. doi: 10.3390/biomedicines9070709 (PMC8301461; doi:10.3390/biomedicines9070709)
Supplement: Supplementary file 1 [file biomedicines-09-00709-s001.zip › Supplementary Materials BOSO D et al.pdf]

biomedicines

MDPI

Supplementary Materials

Porcine decellularized diaphragm hydrogel: a new option for muscle malformations

*Daniele Boso, Eugenia Carraro, Edoardo Maghin, Silvia Todros, Arben Dedja, Monica Giomo, Nicola Elvassore, Paolo De Coppi, Piero Giovanni Pavan, Martina Piccoli**

Figure S1. Muscle-specific proteins in decellularized samples.

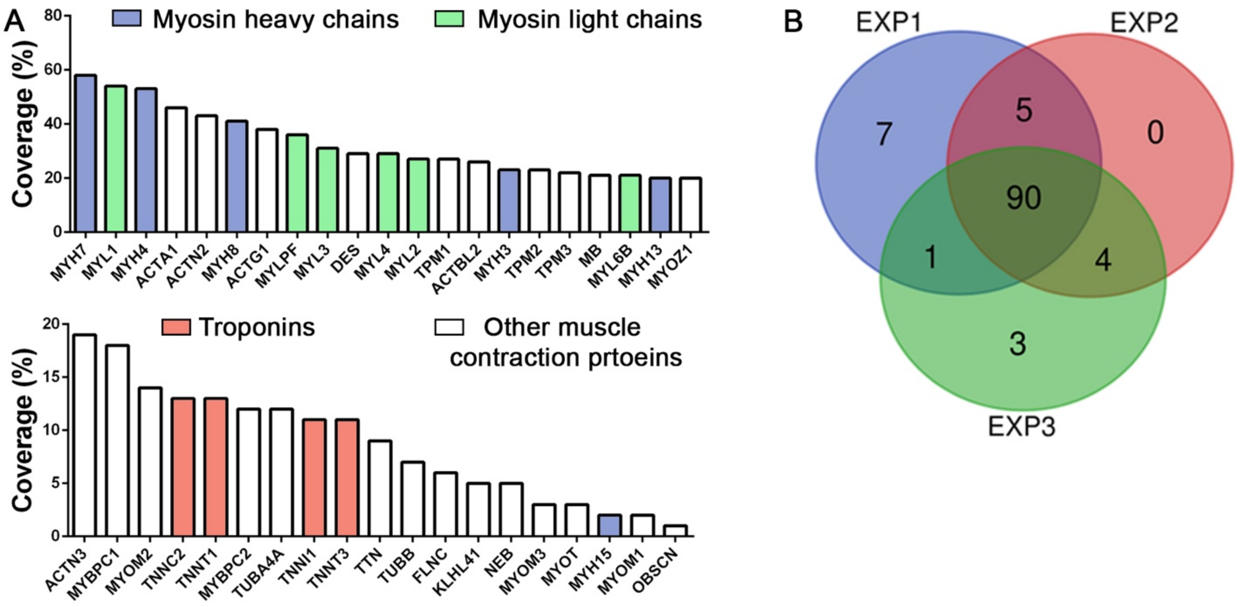

A. Percentage of coverage of specific muscle contraction proteins detected in decellularized samples. Highlighted with colors different isoforms of the same protein. **B.** Venn diagram showing the overlapping of detected proteins among triplicates of three different analyzed samples (EXP = experiment).

Figure S2. 2% w/v dECM-derived hydrogel characterization.

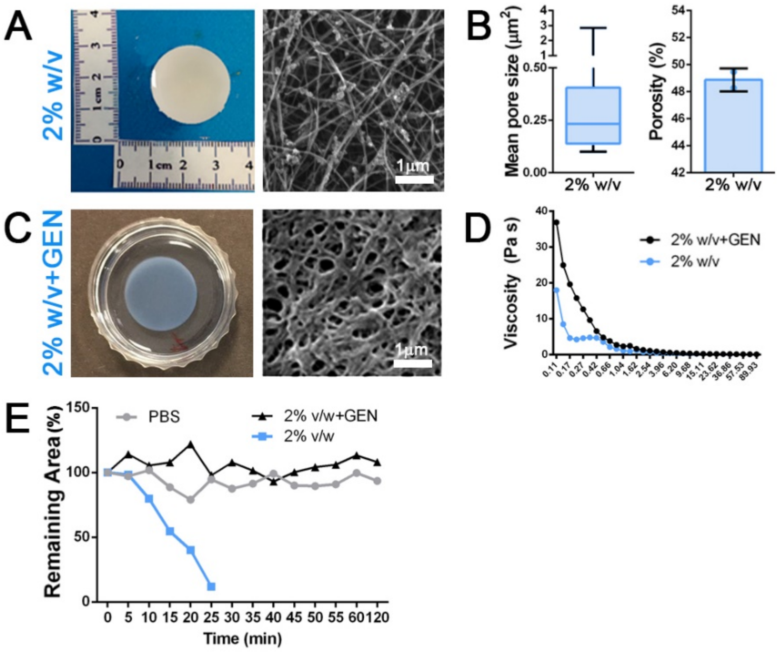

A. Gross appearance and ultrastructure of 2% w/v hydrogels. **B.** Mean pore size and porosity of 2% w/v hydrogels. **C.** Gross appearance and ultrastructure of 2% w/v hydrogels after crosslinking with genipin (+GEN). **D.** Viscoelastic properties under shear stress of 2% w/v hydrogels with and without genipin cross-linking. **E.** Degradation assay of 2% w/v hydrogels with and without genipin crosslinking using collagenase II. PBS: 2% w/v hydrogels without genipin and incubated with PBS (no collagenase II).

Figure S3. Preparation and analysis of crosslinked 3% w/v dECM-derived hydrogel application in ex vivo CDH model.

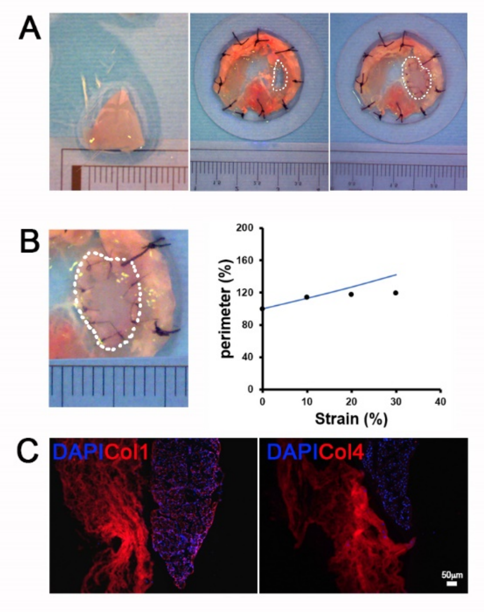

A. Gross appearance of crosslinked 3% w/v hydrogel patch (left panel), diaphragm defect (middle panel) and sutured patch on the defect (right panel). **B.** Calculated perimeter increasing during continuous stimulation up to 30% strain. Dots represent experimental results and solid curve is obtained from FE analysis. **C.** Immunofluorescence staining for the detection of Col1 and Col4 in crosslinked patches and damaged diaphragms after continuous mechanical stimulation.

Figure S4. In vitro hydrogel biocompatibility.

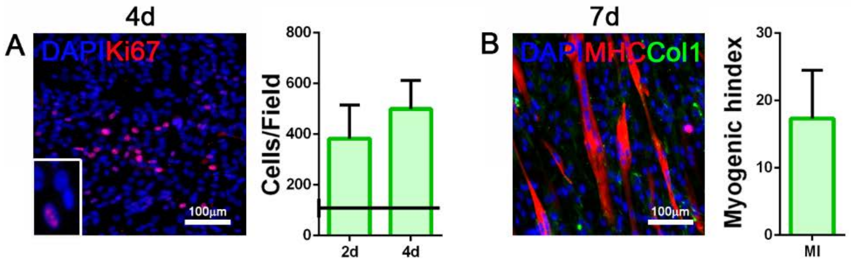

A. 2D culture of mixed hSKMC and hFb population after well coating with dECM-derived hydrogel. Quantification of cell growth (black line = number of seeded cells) after 2 and 4 days of culture. Proliferation marker (Ki67) in red. **B.** Differentiation of seeded cells and quantification of myogenic potential with calculation of myogenic index. Myosin heavy chain (MHC) in red; collagen 1 (Col1) in green. Nuclei are counterstained with DAPI (blue).

Table S1. List of proteins found in dECM with mass spectrometry.

Accession	Description	GENE	Coverage [%]	Peptides [#]
A0A5G2R3E4	Acyl-CoA dehydrogenase very long chain	ACADVL	5	2
P16276	Aconitate hydratase	ACO2	10	5
A0A5S6G831	Actin, alpha skeletal muscle	ACTA1	46	14
A0A287A4R1	Actin beta like 2	ACTBL2	26	6
A0A287AA77	Actin Gamma 1	ACTG1	38	11
F1RHL9	Alpha-actinin-2 isoform 1	ACTN2	43	31
A0A5G2RET9	Actinin Alpha 3	ACTN3	19	15
A0A286ZQ79	Adenylate kinase isoenzyme 1	AK1	10	2
A0A287BFY0	Fructe-bisphphate aldolase	ALDOA	29	9
A0A287B8Z2	Fructe-bisphphate aldolase	ALDOC	12	3
A0A286ZJV6	Annexin	ANXA2	8	2
A0A287B5C0	CMP/dCMP-type deami-nase domain-containing protein	APOBEC2	20	2
A0A5G2R940	Calcium-transporting ATPase	ATP2A1	27	21
P11607	Sarcoplasmic/endoplasmic reticulum calcium ATPase 2	ATP2A2	19	16
A0A287BBS4	ATP synthase subunit alpha	ATP5F1A	32	13
K7GLT8	ATP synthase subunit beta	ATP5F1B	38	14
A0A287AHM1	ATP synthase subunit gamma	ATP5F1C	15	4

Q9GKQ6	biglycan	BGN	5	2
Q5S1S4	Carbonic anhydrase 3	CA3	27	6
F1RJW7	Calsequestrin	CASQ1	13	3
Q5XLD3	Creatine kinase M-type	CKM	11	3
A0A286ZQI3	Creatine kinase	CKMT2	15	5
A0A5G2QQE9	Collagen alpha-1(I) chain	COL1A1	39	34
F1SFA7	Collagen alpha-2(I) chain	COL1A2	38	23
A0A286ZWS8	Collagen alpha-1(II) chain	COL2A1	12	9
A0A286ZQ85	Collagen alpha-1(III) chain	COL3A1	17	15
A0A5G2QW87	Uncharacterized protein	COL6A1	10	6
I3LQ84	Collagen aplpha-2(VI) chain	COL6A2	4	2
I3LUR7	Collagen aplpha-3(VI) chain	COL6A3	4	7
F1S902	Cartilage oligomeric matrix protein	COMP	7	3
A0A287ATJ4	Alpha(B)-crystallin	CRYAB	32	7
P02540	Desmin	DES	29	10
A0A286ZNV2	Elongation factor 1-alpha 2	EEF1A2	19	7
I3LII3	Tr-type G domain-containing protein	EEF2	3	2
Q1KYT0	Beta-enolase	ENO3	34	8
A0A286ZRJ3	Electron transfer flavoprotein subunit alpha	ETFA	8	2
A0A287AAL6	four and a half LIM domains 1	FHL1	24	9
F1SMN5	Filamin-C isoform X4	FLNC	6	10
F1S6B5	Fibromodulin	FMOD	9	3

F1SS24	Fibronectin	FN1	8	12
A0A287BG23	Glyceraldehyde-3-phphate dehydrogenase	GAPDH	40	8
F1RM74	Glyceraldehyde-3-phphate dehydrogenase	GAPDHS	11	3
A0A287BH33	Aspartate aminotransferase	GOT2	18	5
A0A5G2QML3	Histone H2B	H1-3	8	2
P00348	Hydroxyacyl-coenzyme A dehydrogenase	HADH	13	2
Q29554	Trifunctional enzyme subunit alpha	HADHA	5	2
F1SDN2	Acetyl-CoA Acyltransferase	HADHB	6	3
A0A5G2QRW3	Hemoglobin subunit beta	HBB	33	7
F1RII6	Hemoglobin subunit epsilon	HBE1	18	2
A0A5S6G3Y8	Heat shock 27 kDa protein	HSPB1	12	4
A0A287AQR8	SHSP domain-containing protein	HSPB6	36	4
A0A286ZYE6	Isocitrate dehydrogenase	IDH2	25	9
A0A287AIE4	Kelch Like Family Member 41	KLHL41	5	2
F1S911	60S ribomal protein L40	KXD1	8	2
A0A286ZXT7	L-lactate dehydrogenase	LDHA	25	8
F1SR05	L-lactate dehydrogenase	LDHB	7	2
F1RGX4	GLOBIN domain-containing protein	LOC100737768	54	4
F1SCU3	Matrilin 3	MATN3	5	2
P02189	Myoglobin	MB	21	3
A0A5G2RGL7	Malate dehydrogenase	MDH2	34	9

A0A287B5J2	Myosin Binding Protein C1	MYBPC1	18	17
A0A5G2QJC1	Myosin binding protein C2	MYBPC2	12	12
F1SS66	Myosin heavy chain 13	MYH13	20	41
I3L675	Myosin Heavy Chain 15	MYH15	2	5
A0A5G2QTZ6	Muscle Embryonic Myosin Heavy Chain	MYH3	23	53
Q9TV62	Myosin-4	MYH4	53	109
P79293	Myosin-7	MYH7	58	115
A0A5G2R196	Myosin-2	MYH8	41	83
A0A286ZVM3	Myosin light chain 1	MYL1	54	9
Q8MHY0	myin regulatory light chain 2	MYL2	27	3
F1SNW4	Myosin light chain 3	MYL3	31	5
F1RRT2	Myosin light chain 4	MYL4	29	3
A0A5G2QUW6	Myosin Light Chain 6B	MYL6B	21	5
A0A5G2R327	Myosin light chain, phosphorylatable, fast skeletal muscle	MYLPF	36	6
A0A480KXA1	Myomesin-1 isoform a	MYOM1	2	3
A0A286ZK06	Myomesin 2	MYOM2	14	8
A0A287ANE3	Myomesin 3	MYOM3	3	3
A0A287AJY7	Myotilin	MYOT	3	2
Q4PS85	Myozenin-1	MYOZ1	20	3
A0A287BN18	Nebulin	NEB	5	20
A0A287AQG1	Proton-translocating NAD(P)(+) transhydrogenase	NNT	4	3

A0A5G2QZ79	Obscurin, Myosin Light Chain Kinase	OBSCN	1	2
A0A286ZIJ9	ATP-dependent 6-phphofructokinase	PFKM	8	5
B5KJG2	Phphoglycerate mutase 1	PGAM2	9	3
F1RPH0	Phphoglycerate kinase	PGK1	8	2
A0A287A7R4	Pyruvate Kinase M1/2	PKM	11	9
A0A287B9H1	Plectin	PLEC	2	4
A0A287AT94	Alpha-1,4 glucan phphorylase	PYGB	9	6
A0A287B6I2	Alpha-1,4 glucan phphorylase	PYGM	23	28
P16960	Ryanodine receptor 1	RYR1	1	2
A0A287BGP7	Solute carrier family 25 member 3	SLC25A3	6	2
A0A286ZIE8	Solute carrier family 25 member 4	SLC25A4	24	5
P20112	Sparc	SPARC	8	3
F1RK48	Sarcalumenin isoform X1	SRL	7	4
F1SNZ7	Succinate--CoA ligase	SUCLG1	6	2
P02587	Troponin C, skeletal muscle	TNNC2	13	2
A0A286ZI93	Troponin I	TNNI1	11	2
A0A5G2R687	Troponin T, slow skeletal muscle	TNNT1	13	3
A0A5S6I3K1	Troponin T, fast skeletal muscle	TNNT3	11	3
A0A287BHM1	Tropomyosin alpha-1 chain	TPM1	27	10
A0A287AN33	Tropomyosin 2	TPM2	23	10

A0A287BDL6	Tropomyosin alpha-3 chain	TPM3	22	8
A0A5G2QM05	Titin	TTN	9	4
A0A5G2R655	Tubulin alpha 4a	TUBA4A	12	5
F2Z5B2	Tubulin beta chain	TUBB	7	2
F1RPD2	Cytochrome b-c1 complex subunit 2, mitochondrial	UQCRC2	9	2
A0A5S6HVC8	Voltage-dependent anion-selective channel protein 1	VDAC1	22	6
Q9MZ15	Voltage-dependent anion-selective channel protein 2	VDAC2	10	2
A0A5G2QH97	Voltage-dependent anion-selective channel protein 3	VDAC3	19	5
A0A5S6H025	Vimentin	VIM	5	2

Table S2. List of primary and secondary antibodies used.

Name	Source	Dilution
Laminin	Sigma-Aldrich	1:200
Phalloidin (F-actin)	Abcam	1:1000
Col1	Abcam	1:100
Col4	Abcam	1:200
Ki67	Abcam	1:100
MHC	R&D Systems	1:50
Anti rabbit IgG (H+L)-Alexa 594	Invitrogen	1:200
Anti mouse IgG (H+L)-Alexa 594	Invitrogen	1:200
Anti mouse IgG (H+L)-Alexa 548	Invitrogen	1:200
